# Blunting type 1 insulin-like growth factor receptor expression exacerbates neuronal apoptosis following hypoxic/ischemic injury

**DOI:** 10.1186/1471-2202-12-64

**Published:** 2011-06-30

**Authors:** Wen Liu, Joseph A D'Ercole, Ping Ye

**Affiliations:** 1 Department of Pediatrics, University of North Carolina at Chapel Hill, NC 27599-7039, USA

## Abstract

**Background:**

Abundant experimental data have implicated an important role for insulin-like growth factor (IGF) in protecting neuronal cells from injury, including hypoxia/ischemia (H/I) injury, a major cause of neuron death. While the specific interaction of IGFs with neuronal or glial type 1 IGF receptors (IGF1R) has been shown to be essential to IGF actions during development, the same has not been directly demonstrated following H/I injury. To directly examine the role of neuronal IGF1R following H/I injury, we utilized conditional mutant nes-*igf1r*^*-/Wt *^mice and determined the impact of IGF1R haplodeficiency specifically in nestin-expressing neuronal precursors and their progeny on H/I-induced neuronal damage and apoptosis in hippocampus.

**Results:**

H/I induced significant damage to the cerebral hemisphere and hippocampus ipsilateral to the ligated right common carotid artery both in control and nes-*igf1r*^*-/Wt *^mice at postnatal day 10. Blunting IGF1R expression, however, markedly exacerbated H/I-induced damage and appeared to increase mortality. In the ipsilateral hemisphere and hippocampus, nes-*igf1r*^*-/Wt *^mice had infarct areas double the size of those in controls. The size of the ipsilateral hemisphere and hippocampus in nes-*igf1r*^*-/Wt *^mice were 15% to 17% larger than those in controls, reflecting more severe edema. Consistent with its effects on infarct area, IGF1R haplodeficiency causes a greater decrease in neurons in the ipsilateral hippocampus of nes-*igf1r*^*-/Wt *^mice. The reduction in neurons was largely due to increases in neuronal apoptosis. Judged by pyknotic nuclei, TUNEL and caspase-3 labeling, nes-*igf1r*^*-/Wt *^mice had significantly more apoptotic cells than that in controls after injury. To determine possible mechanisms of IGF1R actions, the mRNA expression of the pro-survival proteins IAP-1 and XIAP was determined. Compared to controls, the abundance of cIAP-1 and XIAP mRNA was markedly suppressed in mice with blunted IGF1R or IGF-I expression, while was increased in the brain of IGF-I overexpressing transgenic mice.

**Conclusion:**

IGF1R in neuronal cells is critically important for their survival following H/I injury, and IGF-upregulated expression of neuronal cIAP-1 and XIAP likely in part contributes to IGF-IGF1R protection against neuronal apoptosis following H/I injury.

## Background

Hypoxia/ischemia (H/I) during prenatal brain development is a major cause of neural cell loss, and consequently morbidity and mortality in infants and children [1]. It is estimated that 0.1% - 0.2% of full-term infants and ~50% of surviving preterm infants suffer brain damage caused by H/I injury [2], leading to cerebral palsy, epilepsy, cognitive deficits and growth retardation in affected children.

Several lines of experimental evidence implicate insulin-like growth factor (IGF) in protecting neurons from injury-induced cell death and in promoting neural repair during recovery: 1) IGF-I mRNA abundance falls sharply in injured and surrounding areas during the first 24 hr following H/I injury [3-5], concurrent with significant neuronal apoptosis during this time [6]; and then increases during recovery [4,5]. 2) Injection of exogenous IGF-I into the lateral ventricle immediately or shortly after H/I injury attenuates H/I-induced brain damage and neuron loss [7-9], and oligodendrocyte precursor damage [10]. Locally administered IGF-I also promotes neural tissue recovery [8,9,11]. 3) More recently, peripherally administered IGF-I (through subcutaneous injection [12] or nasal insufflation [13]) mitigates H/I brain injury significantly, in part by promoting the survival of neuronal cells and the proliferation of neural precursor cells. Notably, IGF-I remains effective when given by subcutaneous injection 24 and 48 hrs after H/I injury [12], a finding that supports its potential clinical utility in treating ischemic neural injury.

The type 1 IGF receptor (IGF1R) is essential in mediating IGF actions during neural cell development [14-16]. Whether the IGF1R also plays a key role in IGF's neuroprotection and/or promotion of neural regeneration following H/I injury, however, has not been directly demonstrated. In an earlier study, the data of Guan et al [17] raised the possibility that these IGF-I actions involve mechanisms in addition to those mediated by the IGF1R. des-IGF-I, an IGF-I analog lacking the N-terminal three peptides, retains the high affinity of the native peptide for the IGF1R, but has greatly reduced affinity for IGF binding proteins (IGFBPs). Consequently, it is generally more potent than native IGF-I, because its actions are not inhibited by IGFBPs. Nonetheless, Guan et al. [17] showed that des-IGF-I was much less effective than native IGF-I in mitigating H/I-induced brain damage, suggesting that IGF-I could exert its effects independent of IGF1R and/or that it requires IGFBPs.

To directly address the role of the IGF1R in neuroprotection following H/I, we studied neuronal cell survival following H/I in conditional mutant mice in which the IGFIR expression is halved specifically in nestin-expressing neuronal precursors and their progeny (nes-*igf1r*^*-/Wt *^[15]). We demonstrated that signaling through IGF1R is critically important for neuronal cell survival in developing brains following H/I injury.

## Results

### General

**Figure 1:**
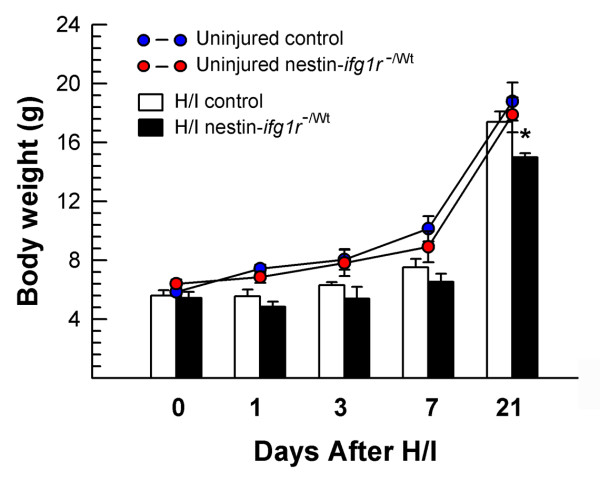
**Body growth of nes-*igf1r***^***-/Wt***^**and control mice following H/I injury**. Values represent mean ± SE from 6 - 15 mice in each group. *, *p *< 0.05, compared to H/I-injured control mice.

Similar to our previous report [15], the somatic growth of uninjured nes-*igf1r*^*-/Wt *^and control mice was not significantly different during development. After H/I injury, however, both nes-*igf1r*^*-/Wt *^and control mice exhibited reduced body weight, being ~80% and ~70% of uninjured mice from 1 to 3 days after injury (DAI) and at 7 DAI, respectively (Figure 1). Somatic growth of injured control mice gradually caught up such that by 21 DAI their body weights were similar to those of uninjured mice. The body weight of injured nes-*igf1r*^*-/Wt *^mice, however, remained reduced by ~15% (Figure 1). A similar percentage of control mice and *nes-igf1r*^-/Wt ^mice (~10%) died during ischemia surgery and hypoxia. Nes-*igf1r*^*-/Wt *^mutant mice, however, appeared to be more susceptible to death after H/I injury. About 16% of nes-*ifg1r *^*-/Wt *^mice died within 48 hr after surgery, while only ~8% of control mice died during the same period (Table 1). The increase, however, did not meet statistical significance.

**Table 1:** The number of mice that die during hypoxia/ischemia (H/I) and within 48 hr following H/I insult.

		Number of Mice That Died		
	
	During Surgery	During Hypoxia	Within 48 hours following H/I	Total death
Control	5 (6.02%)	4 (4.82%)	7 (8.43%)	16 (19.28%)
nes-*igf1r*^*-/Wt*^	2 (6.67%)	3 (6.67%)	7 (15.56%)	12 (26.67%)

Control mice (n = 83) and nes-*igf1r*^*-/Wt *^mice (n = 45) were subjected to H/I insult, as described in the Methods section. The percentage of mice dying during common carotid artery ligation surgery, hypoxia treatment, or 48 hours after H/I insult in each group is reported in the parentheses. Statistical analysis was done using *X*^*2*^, assisted with the software SigmaStat for Windows (SPSS, Inc., Chicago, IL). No significance was observed.

### Blunting neuronal IGF1R expression increases H/I-induced brain edema

**Figure 2:**
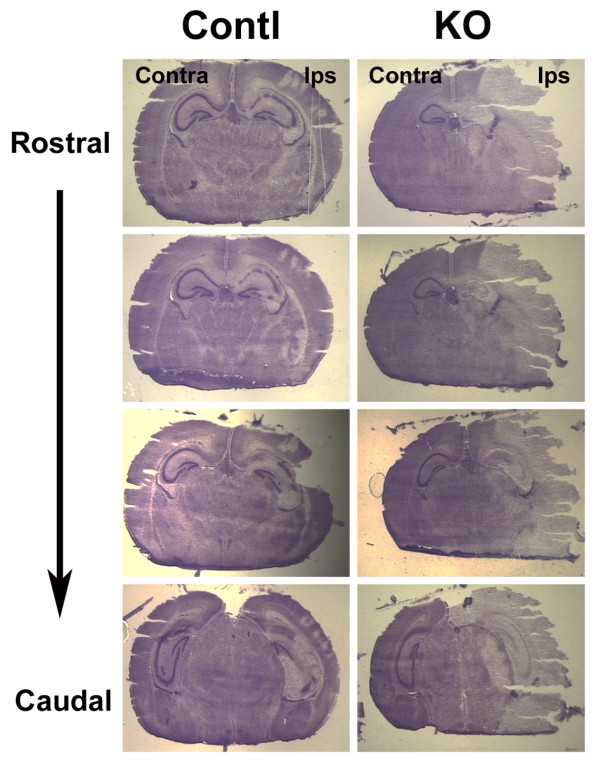
**Representative Cresyl Violet staining of brain sections following H/I injury**. A brain of a nes-*igf1r*^*-/Wt *^mouse (KO, right panels) and its littermate control (Contl, left panels) at 1 DAI were serially frozen-sectioned and stained with Cresyl Violet. Contra = contralateral, and Ips = ipsilateral. Note that significant damage in hemisphere ipsilateral to the right ligated common carotid artery.

H/I-induced edema, characterized by increased tissue water, enlarged tissue volume (swelling), and reduced cell density, has been well-documented [18-20]. Similar to the morphologic observation of Menley et al. [21], our examination of brains of injured mice showed that the hemisphere and hippocampus ipsilateral to the ligated right common carotid artery appeared to be larger than their contralateral counterparts, while cell density in injured areas was significantly reduced (Figure 2, data not shown). This increased size caused by H/I is consistent with H/I-induced swelling/edema. To quantify H/I-induced swelling/edema in hemisphere and hippocampus, we performed stereological analysis of rapidly frozen brains of H/I-injured mice at 1 DAI.

While the size of the ipsilateral hemisphere was not significantly increased in control mice, it was significantly increased in nes-*igf1r*^*-/Wt *^mice (Table 2). Consistently, the size of the ipsilateral hippocampus, including both hippocampal proper (HIP) and dentate gyrus (DG), also was increased in control and nes-*igf1r*^*-/Wt *^mice. When calculated as a percentage of their respective contralateral sizes, the volumes of the ipsilateral hemisphere and hippocampus in nes-*igf1r*^*-/Wt *^mice were increased by 15% to 17%, as compared to that of control mice (Table 2).

**Table 2:** Volume of hippocampus in nes-*igf1r*^*-/*^^*W*^^*t *^and control mice at 1 DAI (mm^3^, mean ± SE).

	Control Mice		**nes-*igf1r***^***-/Wt***^	
		
	Contralateral	Ipsilateral	Contralateral	Ipsilateral
Hemisphere	18.59 ± 0.50	19.64 ± 0.47	13.67 ± 0.37	16.71 ± 0.93*
Hippocampus	2.29 ± 0.21	2.67 ± 0.26#	1.04 ± 0.52	1.41 ± 0.11**

Control mice (n = 8) and nes-*igf1r*^*-/Wt *^mice (n = 6) that underwent H/I were sacrificed at 1 DAI and their brains were frozen-sectioned. The volumes of hemisphere and hippocampus (including both HIP and DG) from each side were measured. #, *p *< 0.1; *, *p *< 0.05; **, *p *< 0.02, compared to their respective contralateral.

### Blunting neuronal IGF1R expression exacerbates H/I-induced infarct

**Figure 3:**
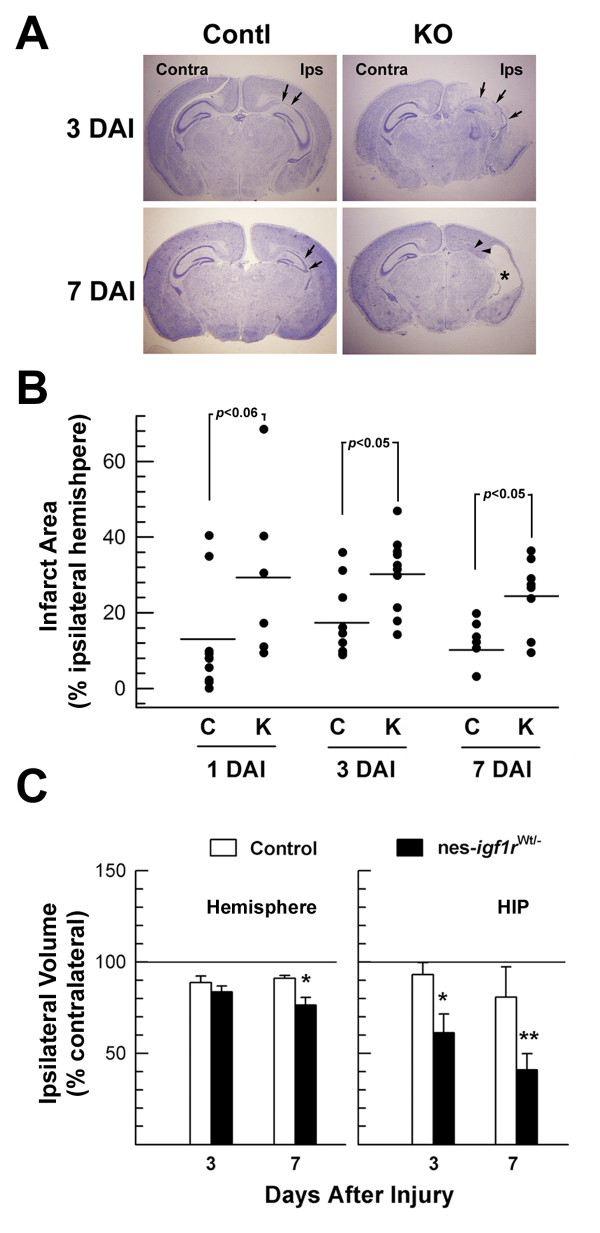
**Increased infarct size and reduced hemisphere volume in injured nes-*igf1r***^***-/Wt***^**mice**. Paraffin-embedded brains were serially sectioned and stereologically analyzed as described in Methods. **A**. Representative Cresyl Violet staining of brain sections from a nes-*igf1r*^*-/Wt *^mouse (KO) and its littermate control (Contl) at 3 and 7 DAI, respectively. **B**. Infarct size. The area of infarct in the ipsilateral hemisphere is expressed as a percentage of the ipsilateral hemisphere area. Each dot represents the value from an individual mouse. Values represent mean ± SE from 6 - 10 brains in each group. **C**. Quantification of hemisphere and hippocampus volume. Hemisphere and hippocampus volume is expressed as percentage of contralateral counterparts. Values represent mean ± SE from 5 - 8 brains in each group. *, *p *< 0.05; **, *p *< 0.01, compared to control mice.

While hypoxia and ischemia without hypoxia had no obvious impact on neuronal cells in both control mice and nes-*igf1r*^*-/Wt *^mice, ischemia followed by hypoxia resulted in significant and widespread damage to the hemisphere ipsilateral to the ligated right common carotid artery, including neurons in cerebral cortex, HIP and DG (Figures 2 and 3A). H/I of control mice resulted in an infarct area that averaged ~13% and ~16% of the ipsilateral hemisphere at 1 DAI and 3 DAI, respectively (Figure 3B). Compared to control mice, the percentage of ipsilateral hemisphere infarct area in nes-*igf1r*^*-/Wt *^mice was doubled or more at each time point examined (Figure 3B). Infarct area in mice 7 DAI often became cystic or occupied by an enlarged lateral ventricle (Figure 3A). Compared to controls the relative volume of the ipsilateral hemisphere in nes-*igf1r*^*-/Wt *^mice, thus, was significantly smaller at 7 DAI, and that of the hippocampuses was much smaller at both 3 and 7 DAI, (Figure 3C).

### Blunting neuronal IGF1R expression exacerbates H/I-induced reduction of HIP and DG neurons

To more precisely define the impact of reduced IGF1R expression on neuronal cells following H/I injury, we performed stereological analysis of HIP and DG. HIP and DG have a distinct and relatively simple cyto-architecture that facilitates our analysis of neuronal cell response following injury.

**Figure 4:**
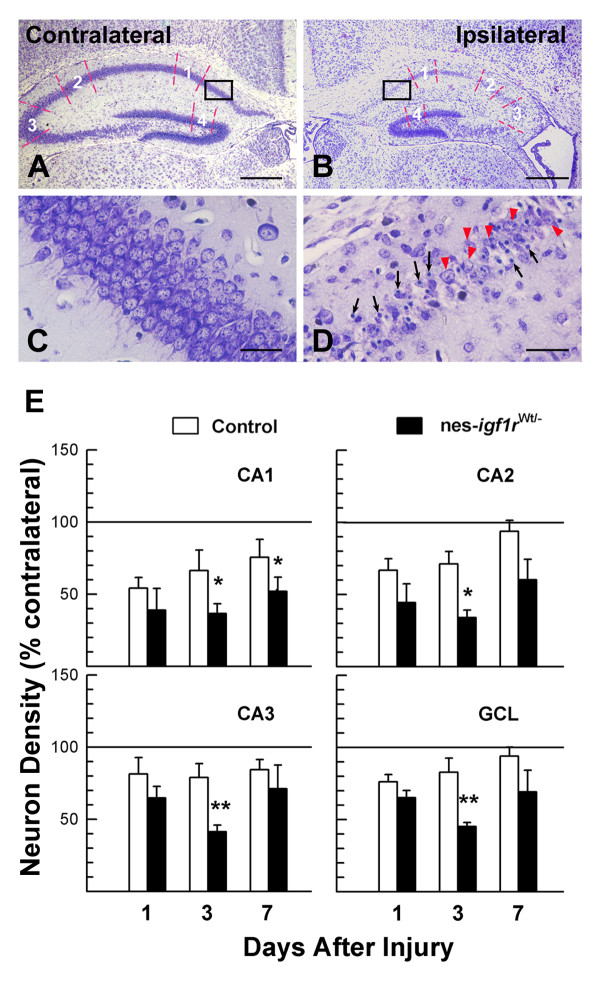
**Decrease in CA and DG neuron density in nes-*igf1r*^*-/Wt*^mice following H/I injury**. **A - D**. Representative microphotographs of contralateral (A and C) and ipsilateral (B and D) HIP and DG of a control mouse at 3 DAI. Panels C and D are high magnification of areas (black rectangle) shown in panels A and B, respectively. White letters and associated red dashed lines in A and B indicate the areas used to measure the density of neurons and pyknotic cells where 1 is the pyramidal neuron layer (PCL) in CA1, 2 is PCL in CA2, 3 is PCL in CA3, and 4 is the granule cell layer (GCL) in DG. In panel D, red arrowheads show healthy appearing neurons and black arrows point to pyknotic cells. Neurons were identified morphologically. ***E***. Quantification of neuron density in the PCL of CA1 to CA3 and in GCL of DG. Values represent mean ± SE from 6 - 10 brains in each group. Neuronal cell density is expressed as a percentage of contralateral. *, *p *< 0.05; **, *p *< 0.01, compared to control mice.

Following H/I, control mice exhibited significant neuronal loss in the injured ipsilateral HIP and DG, resulting in a significant reduction in neuron density in Cornu Ammonis (CA) and DG regions (Figure 4). When compared to the contralateral, neuron density in control mice at 1 DAI exhibited a 35 - 45% reduction in the pyramidal neuron layer (PCL) of ipsilateral CA1 and CA2, and a 20 - 30% reduction in CA3 PCL and in DG granule cell layer (GCL) (Figure 4E). Many neurons in affected ipsilateral CA and GCL contained a condensed cell nucleus (pyknotic cells) (Figure 4D), a hallmark of apoptosis. With increasing time after injury, neural repair and recovery in control mice were apparent as manifested by a gradually increasing neuron density, although neuronal density remained reduced compared to the uninjured contralateral hemisphere (Figures 4, panels C, D and E).

Blunting IGF1R expression in nes-*igf1r*^*-/Wt *^mice resulted in a more dramatic reduction in the density of neurons in both H/I-injured ipsilateral CA PCL and DG GCL. When compared to control mice at 1 DAI, nes-*igf1r*^*-/Wt *^mice exhibited a trend toward a reduction in relative neuron density in ipsilateral CA3 PCL and GCL (by 15%), and in CA1 and CA2 PCL (by 30-34%). At 3 DAI the relative density of neurons was significantly reduced by 45 - 50% in PCL of CA regions and in GCL of DG (Figure 4). At 7 DAI the relative densities of ipsilateral CA and DG neurons in nes-*igf1r*^*-/Wt *^mice remained reduced in CA1.

**Figure 5:**
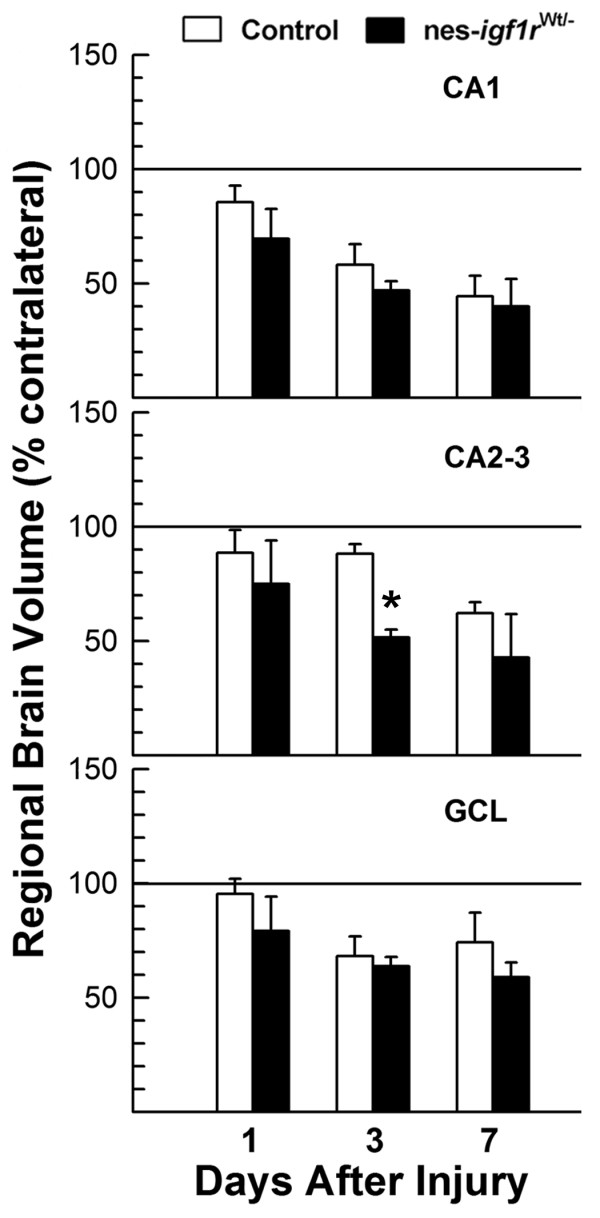
**Decreased volume of CA PCL and DG GCL in nes-*igf1r***^***-/Wt***^**mice following H/I injury**. Volumes of PCL and GCL are expressed as a percentage of the contralateral volume. Values represent mean ± SE from 6 - 10 brains in each group. *, *p *< 0.05, compared to control mice.

Consistent with the reduction in the size of hemisphere and hippocampus (Figure 3C), the volume of PCL and GCL ipsilateral to H/I injury also appear to be reduced (Figure 5). In this experiment, the volume of PCL in CA2 and CA3 was combined for calculation. With increasing time following H/I, the volume of ipsilateral PCL and GCL gradually decreased in both control and nes-*igf1r*^*-/Wt *^mice. Compared to control mice, however, the magnitude of reduction in nes-*igf1r*^*-/Wt *^mice appeared to be greater (Figure 5); but these changes (except for PCL in CA2-3 at 3 DAI) did not meet statistical significance.

### Blunting neuronal IGF1R expression aggravates H/I-induced apoptosis in HIP and DG neurons

Injury-induced cell death largely contributed to the neuronal loss observed in both nes-*igf1r*^*-/Wt *^and control mice. When the histological appearance of pyknosis was used as a measure, more than 90% of the damaged cells appeared to be apoptotic during the course of the experiment. Few neurons in HIP and DG contralateral to the ligated right common carotid artery underwent apoptosis, and the density of pyknotic cells was from 20 - 114 cells/mm^2 ^in control mice and 57 - 361 cells/mm^2 ^in nes-*igf1r*^*-/Wt *^mice, respectively. When compared to control mice during development, the density of pyknotic cells was significantly increased in nes-*igf1r*^*-/Wt *^mice, a finding that is consistent with our previous report [15]. A marked increase in the number of pyknotic cells in nes-*igf1r*^*-/Wt *^mice also was observed when pyknotic cells number was expressed as a percent of total cell number (0.37% - 1.76% in control mice and 2.37% - 7.69% in nes-*igf1r*^*-/Wt *^mice).

**Figure 6:**
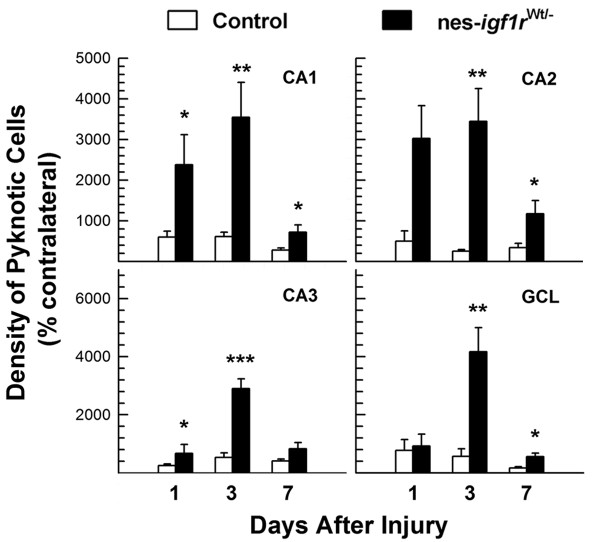
**Increase in pyknotic cell density in nes-*igf1r***^***-/Wt***^**PCL and GCL following H/I injury**. Values represent mean ± SE from 6 - 10 brains in each group. Pyknotic cell density is expressed as percentage of contralateral. *, *p *< 0.05; **, *p *< 0.01; ***, *p *< 0.001, compared to control mice.

Following H/I injury the density of pyknotic cells was markedly increased in the ipsilateral side in both groups of mice at each time assessed. When compared to control mice, the density of pyknotic cells in nes-*igf1r*^*-/Wt *^mice was 1.6 - 5 folds more in CA1 and CA3 at 1 DAI, and 4 - 12 fold more in all regions examined at 3 DAI (Figure 6). While by 7 DAI the number of pyknotic cells in ipsilateral HIP and DG fell significantly in both control and nes-*igf1r*^*-/Wt *^mice, the density of pyknotic cells in nes-*igf1r*^*-/Wt *^mice remained elevated, compared to control mice.

**Figure 7:**
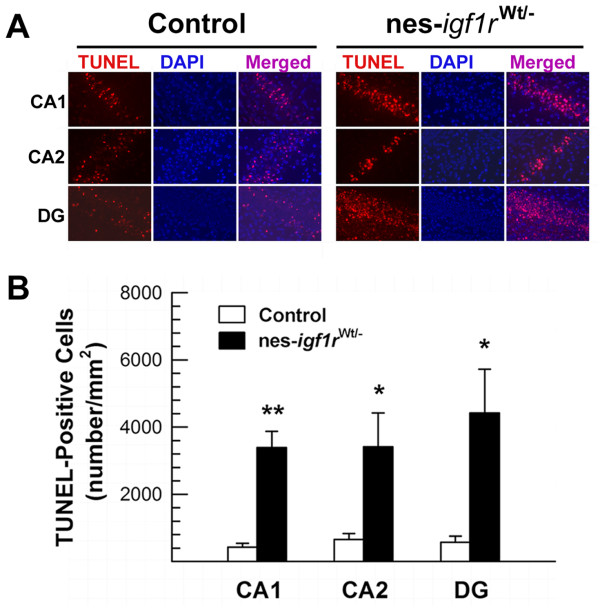
**Increased TUNEL-positive cell density in the PCL and GCL from nes-*igf1r***^***-/Wt***^**mice**. **A**. Representative microphotographs of TUNEL staining in ipsilateral HIP and DG in a nes-*igf1r*^*-/Wt *^mouse and a control mouse at 3 DAI. Note that there are many more TUNEL-positive cells in the ipsilateral hemisphere of the nes-*igf1r*^*-/Wt *^mouse. **B**. Quantification of TUNEL-positive cells in nes-*igf1r*^*-/Wt *^and control mice at 3 DAI. Values represent mean ± SE from 6 - 10 brains in each group. *, *p *< 0.05; **, *p *< 0.01, compared to control mice.

Increased neuronal apoptosis in nes-*igf1r*^*-/Wt *^mice was confirmed using TUNEL labeling of brain sections from mice 3 DAI. Most, if not all, pyknotic cells were found to be TUNEL-positive, and very few TUNEL-positive cells were observed in contralateral hemispheres. However, abundant cells were TUNEL-labeled in the ipsilateral CA PCL and DG GCL in both control and nes-*igf1r*^*-/Wt *^mice (Figure 6). Similar to our findings on pyknotic cells, the density of TUNEL-positive apoptotic cells was increased by 5 - 8 fold in ipsilateral CA PCL and DG GCL of nes-*igf1r*^*-/Wt *^mice (Figure 7B). Quantification of cells positive for active caspase-3 in the hippocampus also showed a similar result (data not shown).

### IGF-I-IGF1R signaling promotes survival protein mRNA expression

**Figure 8:**
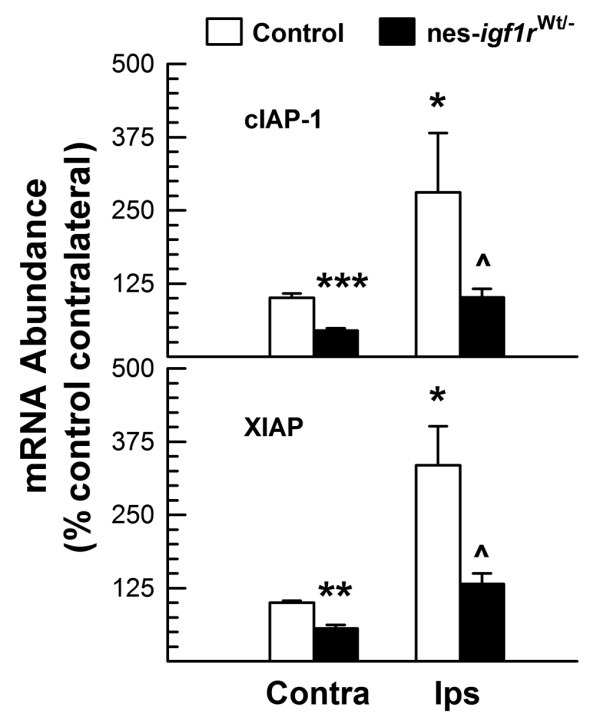
**Reduced expression of XIAP and cIAP-1 mRNA in nes-*igf1r***^***-/Wt***^**mice**. Total RNA from the contralateral (Contra) and ipsilateral (Ips) hemispheres of nes-*igf1r*^*-/Wt *^mice and controls at 1 DAI was isolated and subjected to qRT-PCR analysis. Values represent mean ± SE from 6 - 10 brains in each group. *, *p *< 0.05; **, *p *< 0.01; ***, *p *< 0.001, compared to contralateral hemisphere of control mice. ^, *p *< 0.05, compared to ipsilateral hemisphere of control mice.

To determine whether IGF1R signaling promotes neuronal survival by regulating the expression of anti-apoptosis protein genes, we examined the abundance of mRNA for cellular inhibitor of apoptosis protein (cIAP)-1 and X-linked IAP (XIAP). Compared to that in control mice, the contralateral hemisphere of nes-*igf1r*^*-/Wt *^mice exhibited ~55% and ~40% reductions in the abundance of cIAP-1 and XIAP mRNA, respectively (Figure 8), indicating that blunting IGF1R significantly reduced the abundance of mRNA for cIAP-1 and XIAP in developing brain. Following H/I injury, the abundance of cIAP-1 and XIAP mRNA was increased by ~180% and ~234% in ipsilateral hemispheres of control mice, as compared to that of their contralateral hemispheres. This finding is consistent with data showing that cIAP-1 and XIAP protein abundance is increased in H/I-injured brain [22]. The increases in cIAP-1 and XIAP mRNA abundance induced by H/I injury, however, were suppressed by IGF1R haploinsufficiency in nes-*igf1r*^*-/Wt *^mice (Figure 8). Compared to the respective ~180% and ~234% increases in cIAP-1 and XIAP mRNA in the control ipsilateral hemispheres, the abundance of cIAP-1 and XIAP mRNA in the ipsilateral hemispheres of nes-*igf1r*^*-/Wt *^mice was only increased by ~120% and ~133% (*p *< 0.05), respectively.

**Figure 9:**
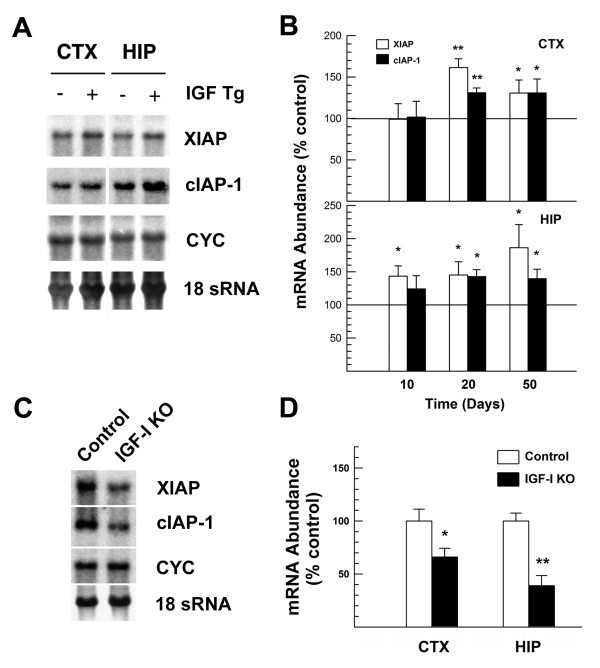
**Altered XIAP and cIAP-1 mRNA expression in mutant mice with altered IGF-I expression**. **A**. Representative Northern blot analysis of XIAP and cIAP-1 mRNA in cerebral cortex (CTX) and hippocampus (HIP) from an IGF-I transgenic mouse and a littermate control at postnatal day (P) 20. IGF-I transgenic mouse is indicated with + at top of lanes. Cyclophilin (CYC) signals and image of methylene blue staining of 18S rRNA (18S rRNA) were shown as bottom two panels, respectively. **B**. Quantification of XIAP and cIAP-1 mRNA in developing CTX and HIP of IGF-I transgenic mice. **C**. Representative Northern blot analysis of XIAP and cIAP-1 mRNA in HIP from a P20 *igf-I*^-/- ^mouse (IGF-I KO) and a control mouse. **D**. Quantification of XIAP and cIAP-1 mRNA in the CTX and HIP P20 *igf-I*^-/- ^mice and their littermate controls. In both panels B and D, values represent mean ± SE from 4 - 6 brains in each group. *, *p *< 0.05; **, *p *< 0.01, compared to control mice.

To directly determine whether IGF-I has a role in the IGF1R signaling regulation of the brain IAP expression, we determined IAP mRNA abundance in IGF-I transgenic mice at 20 days of age, when the IGF-I transgene expression peaks [23]. *igf-I*^*-/- *^mice were also used as a complimentary model. Consistent with the results observed in nes-*igf1r*^*-/Wt *^mice, blunting IGF-I expression in *igf-I*^*-/- *^mutant mice resulted in a significantly reduced abundance of cIAP-1 and XIAP mRNA in HIP and cerebral cortex (CTX) (Figure 9). In contrast, overexpressing of IGF-I in brain markedly increased the abundance of cIAP-1 and XIAP mRNA in HIP and CTX (Figure 9).

## Discussion

Our data strongly indicate that the interaction of IGF and IGF1R in neuronal cells plays an important role in neuronal survival against H/I brain injury. We found that nes-*igf1r*^*-/Wt *^mice with blunted IGF1R expression specifically in nestin-expressing neuronal precursors and their progeny exhibit exacerbated H/I-induced injury. Specifically, compared to normal mice these mutant mice have: 1) worsened brain edema, 2) increased rates in neuronal death, leading to greater decreases in neuron number, and 3) increased infarct area. We also show that a reduction in signaling through IGF1R significantly down-regulates the mRNA expression of cIAP-1 and XIAP, two IAP family proteins that are capable of suppressing cell apoptosis in multiple cell types, including neurons. In contrast, overexpression of IGF-I in brain increases the mRNA expression of cIAP-1 and XIAP. These data suggest that the down-regulated expression of neuronal cIAP-1 and XIAP, caused by reduction in neuronal IGF-IGF1R signaling, contributes to the aggravated neuronal apoptosis and H/I brain injury in nes-*igf1r*^*-/Wt *^mice.

H/I brain injury causes significant brain edema in rats that begins 30 minutes after injury and remains 6 DAI [20]. Consistently, our results show that the ipsilateral ischemic hemispheres and hippocampuses had much larger volumes than the contralateral control hemispheres and hippocampuses, and these acute increases in volume must result from edema. Furthermore, when IGF1R expression was halved in nes-*igf1r*^*-/Wt *^mice, the relative increases in ipsilateral hippocampal volume induced by H/I injury were much larger, and likely represented increases in the severity of edema. This finding further supports an important role for IGF-IGF1R signaling in protecting brain from H/I-induced injury. Because the Cre^nestin ^transgene is not expressed in brain blood vessels (our unpublished data) and brain vascular IGF1R signaling and cytoarchitecture is apparently normal in nes-*igf1r*^*-/Wt *^mice [15], the aggravated brain edema in H/I-injured nes-*igf1r*^*-/Wt *^mice is likely a result of increased responses to more severe neuronal damage. A possible compromise of blood brain barrier secondary to neuronal injury, however, cannot be excluded.

Cell death in injured areas is a hallmark of the H/I-induced brain damage, and usually begins shortly after H/I and continues for several days, reaching its peak 2 - 3 DAI [24,25]. In line with previous reports, we also observed abundant loss of neuronal cells during the first 3 days after injury. Most of the observed dying/dead neuronal cells exhibited characteristics of programmed cell death (i.e., apoptosis), such as condensed nuclei, nuclear DNA fragmentation that can be end labeled, and an increased abundance of active caspase-3. Our data, thus, are consistent with published data [22,24-26], and indicate that under our experimental conditions the majority of dead neuronal cells underwent apoptosis, although necrosis also is likely to contribute to H/I-induced cell death. When compared to control mice, blunting IGF1R expression in nes-*igf1r*^*-/Wt *^mice resulted in 6 - 11 fold more dead cells during the first 3 days after injury. At 7 DAI the number of dead cells observed was still increased but more modestly (by 2 - 3 fold). These results indicate that biallelic IGF1R expression is required for the normal survival of hippocampal neurons after H/I-induced injury, and that IGF1R signaling may be more critical in neuronal survival during the first 3 days after injury.

It is well-documented that the expression of both IGF-I and IGF-II, IGF1R ligands, is temporally and spatially regulated in H/I-injured brain (see reviews by D'Ercole et al. [27] and Popken et al. [28]). Alteration in serum IGFs in subjects with H/I brain injury also has been reported [29,30]. While IGF-I has been widely implicated in neuronal protection and repair following H/I, the functions of IGF-II in H/I brain injury is not clear. Guan and co-workers [31] have shown that administering IGF-II does not reduce cortical infarction induced by H/I, and that co-administration of IGF-II with IGF-I abolishes the protective effects of IGF-I. These data suggest that IGF-II likely has a function(s) that differs from IGF-I. Under our experimental conditions it is not clear whether the IGF1R exerts its neuroprotective effects by interacting with IGF-I, IGF-II or both. Regardless of its ligand, or the origin of these ligands, our data strongly indicate an important role for signaling through IGF1R in neuronal protection following H/I injury.

cIAP-1 and XIAP, two members of IAP family proteins, are capable of suppressing the activity of caspases (the enzymes that are involved in apoptotic destructive processes) by either enhancing caspase degradation (cIAP) or directly inhibiting their activity (XIAP) [32]. The findings that cIAP-1 and XIAP mRNA is increased in IGF-I overexpressing transgenic mice and markedly reduced in nes-*igf1r*^*-/Wt *^mice and IGF-I null mutant mice demonstrate that signaling through IGF1R has an important role in regulating the expression of mRNA for these two proteins during brain development. We further showed that following injury the expression of cIAP-1 and XIAP mRNA is increased in ipsilateral hemisphere, and that these increases are dampened by IGF1R haploinsufficiency. These data suggest that increased cIAP-1 and XIAP, at least in part, mediates the anti-apoptotic effects of IGF signaling in CNS neuronal cells during development and following H/I injury.

## Conclusions

Taken together, our data demonstrated that blunting IGF1R expression specifically in neuronal cells exacerbates H/I-induced injury, and thus, are consistent with the proposition that IGF-IGF1R interaction in neuronal cells is critically important for their survival following H/I injury. In addition, our data showing that IGF-IGF1R signaling up-regulates the expression of neuronal cIAP-1 and XIAP during development and following H/I, strongly suggest that cIAP-1 and XIAP are two candidate targets for therapeutic treatment of H/I-induced brain injuries.

## Methods

### Animals and H/I Injury

Nes-*igf1r*^*-/Wt *^conditional mutant mice, generated as previously described [15], were bred as heterozygotes. As previously reported, *igf1r*^*lox/Wt *^and nestin-Cre transgenic mice have normal postnatal growth, and our preliminary studies show that these mice exhibit similar changes to those of wildtype (Wt) mice in responding to H/I injury. Thus, these mutant mice were grouped and considered controls. Transgenic mice that express a human IGF-I cDNA driven by a metallothionein-I promoter (IGF-I transgenic mice) [23], and mice with a global IGF-I null mutation (*igf-I*^*-/- *^[14,33] also have been described elsewhere. All procedures used were consistent with the guidelines of the National Institutes of Health and approved by the institutional review committees of the University of North Carolina at Chapel Hill.

H/I injury was done using a modified method [34,35] that was adapted from a neonatal rat method [18]. Briefly, mice at postnatal day 10 (weighting 4 to 5 grams) were subjected to artery ligation surgery or sham surgery under isoflurane anesthesia (4% for induction and 1.5-2% for maintenance). After the skin was sterilized with 75% ethanol and 10% povidone-iodine, a midline neck incision (0.5 - 1 cm in length) was made, and the right common carotid artery was carefully isolated and ligated with a bipolar coagulator. The wound was then closed with interrupted nylon sutures. During the procedure, which typically took no longer than 10 minutes, body temperature was maintained using a heating lamp. After ligation or sham surgery, mice were allowed to recover from anesthesia in a chamber partially submerged in a 37°C water bath. After ~10 minutes, mice were returned to their dam. One hour after surgery, mice were individually placed in a chamber that was partially submerged in a water bath (36 ± 0.2°C), subjected to hypoxia (10% O_2_, balanced with N_2_) for 45 minutes, and then were returned to their dam. Both control mice and nes-*igf1r*^*-/Wt *^mice resumed normal activity and feeding within 5-10 minutes after return to their dam.

Mice were sacrificed 1, 3 or 7 DAI. Similar to previously reported data [18,36], control mice or nes-*igf1r*^*-/Wt *^mice, which underwent hypoxia alone, ischemia alone, sham surgery, or sham surgery followed by hypoxia, exhibited similar body growth, behavioral and brain morphological characteristics as their intact untreated counterparts. H/I injury resulted in a suboptimal somatic growth (see Results and Figure 1). As we previously reported, reduction in IGF1R expression causes brain growth retardation [15], and therefore, all changes are expressed as% of the contralateral brain hemisphere, except where indicated.

### Histology and Stereological Analysis

After being fixed by submersion in 4% paraformaldehyde overnight, brains were paraffin-embedded, and coronally sectioned at 8 μm in thickness. Two to three sets of serial sections that comprised very 10^th ^section were collected. To estimate the volume of brain regions, one series of sections were stained with cresyl violet, and the area of brain regions on each section (corresponding to plates between number 41 and 52 [37]) was measured under a microscope, assisted with Stereo Investigator software (Microbrightfield, Colchester, VT). The volume (V) was then estimated using the formula: V = ΣA × T × I, where ΣA = sum of area measured on each section, T = section thickness, and I = section intervals.

To determine the density of pyramidal neurons in CA PCL regions and granule neurons in DG GCL, stained section corresponding to plate 50 [37] from each brain was selected, and cell nuclei within delineated areas were counted, assisted with Stereo Investigator software. In each section the cell density in each brain region was calculated by dividing the regional cell count by its respective area.

To determine infarct area, sections corresponding to plate 50 [37] from each paraffin-embedded brain were stained with cresyl violet. The area of infarct in the ipsilateral hemisphere was then measured using Stereo Investigator, as described above.

To evaluate possible brain swelling/edema after H/I injury, some brains were fresh-frozen in liquid N_2 _to preserve water content and brain structures. Serial sections (20 μm in thickness) were cut coronally on a cryostat. Two to three sets of serial sections that comprised every 6^th ^section were collected. One series of sections (also corresponding to plates between number 41 and 52) were fixed with 4% paraformaldehyde, and stained with cresyl violet. The areas of ipsilateral and contralateral hemisphere and hippocampus were measured, and their volumes were calculated as described above.

### Terminal uridine nucleotide end labeling (TUNEL)

After paraffin-sections (corresponding to plate [37]) were dewaxed and rehydrated, TUNEL-positive apoptotic cells were detected using an *In Situ *Cell Death Detection Kit (Roche Applied Science, Indianapolis, IN), following the manufacturer's protocol. The sections were then counterstained with the nuclear dye 6-diamidine-2'-phenylindole dihydrochloride (DAPI, Invitrogen, Carlsbad, CA). Images of TUNEL-positive cells within CA and DG regions were digitally captured using a microscope and a Spot Jr. digital camera (Diagnostic Instruments, Sterling Heights, MI). Within delineated areas (measured and assisted with a Stereo Investigator software), TUNEL-positive cells were counted, and then their densities were calculated.

### Quantitative Real Time-PCR (qRT-PCR) and Northern Blot Hybridization Analysis

Slides containing coronal frozen-sections (20 μm in thickness, and corresponding to plate 48 - 50) were briefly air-dried. Contralateral and ipsilateral hemisphere tissues were scraped from the slides and collected, and total RNA was isolated using a RNA plus kit (Qiagen, Valencia, CA). cDNA reverse transcription was performed, and the resultant mRNA-derived cDNA was quantified by qRT-PCR using specific primer sets. The sequences of the primers used are as follows: cIAP-1 (Genbank accession number: BC145985), forward primer: 5'-gatggtggcttgagatgttgg-3', reverse primer, 5'-tttctccagggccaaaatgc-3'; XIAP (Genbank accession number: NM_009688), forward primer, 5'-ctattggatgagaaggggcaag-3', reverse primer, 5'-atagatagctgctcccggatg-3'. Primers for 18S rRNA were obtained from Invitrogen. The abundance of mRNA was determined, based on a standard curve for each target mRNA and 18S rRNA, as described [38].

Northern blot hybridization and quantification of mRNA abundance on blots were performed as previously described [23]. Total RNA was isolated using the acidic guanidinium thiocyanate-phenol-chloroform method [39]. ^32^P-labeled single stranded cDNA probes were generated as previously described [23], using plasmid containing XIAP (also known as MIHA) or cIAP-1 (also known as BIRC2 or MIHB) cDNA. XIAP and cIAP-1 plasmids were generously provided by Dr. Uren [40]. PCR-amplified XIAP and cIAP-1 cDNA fragments correspond to base pairs (bp) 584-870 (Genbank accession number: U36842) and bp 1685-1898 (Genbank accession number: U37547), respectively.

### Statistics

All experiments were repeated at least two times. Student-*t *test or one-way ANOVA was used to test statistical significance among the groups, followed by comparison of each group mean with the Newman-Keuls-Student test assisted with the software SigmaStat for Windows (SPSS, Inc., Chicago, IL).

## Competing interests

The authors declare that they have no competing interests.

## Authors' contributions

PY and AJD designed the study. WL and PY performed the experiments and collected data. PY and AJD analyzed the data and wrote the manuscript. All authors read and approved the manuscript.
